# Incidences of nosocomial infection in Uruguayan adult intensive care unit 2010

**DOI:** 10.1186/1753-6561-5-S6-P77

**Published:** 2011-06-29

**Authors:** SE Guerra, H Albornoz, R Rosa, M Godino, T Camou, A Galiana, S Martinez, G Rios, G Rehermann, H Bagnulo

**Affiliations:** 1 Infection Control Committee, Ministry of Public Health, Montevideo, Uruguay

## Introduction / objectives

ICU patients (ptes) account for a large proportion of HI. Its epidemiology allow us to identify key issues and prioritize interventions. We determined the incidence of HI in ICU in Uruguay and described specific locations, dispositive-associated rates and microorganisms.

## Methods

A national surveillance system for HI was implemented in 2006; prospective surveillance was performed using NNISS criteria. It is mandatory, to record online and send their data to the Ministry of Health. Data are audited and results published annually.

## Results

We present 2010 results of medical-surgical ICUs. 53 hospitals reported. 13611 ptes were surveyed (99541 ptes-days). 2340 HI episodes were reported (density of incidence rate (ID) 23.5/1000 ptes-days and cumulative incidence 17.2%). Ventilator-associated pneumonia (VAP) (34%), bronchitis (B) (28%), catheter-associated urinary tract infection (CAUTI) (21%) and central-line associated bacteremia (CLAB) (8%) were the main infectious sites. VAP ID was 14.2/1000 ventilator-days (dispositive utilization (DU) 0.5, secondary bacteremia (SB) 5.3% and contributor mortality (CM) 19.7%), CLAB ID was 1.9/1000 catheter-days (DU 0.7, (CM) 17.7%) and CAUTI ID was 5.8/1000 catheter-days (DU 0.8, (SB) 3.7%, (CM) 5.1%). Microorganisms were *S. aureus* (19, 17, and 11.4% in VAP, B and CLAB), *A. baumanii* (VAP 22%, B 18%), *Ps. aeruginosa* (16, 19, 6.4 and 10% in VAP, B, CLAB and CAUTI) and *K. pneumoniae* (12, 13, 14.9 and 14% in VAP, B, CLAB and CAUTI).

## Conclusion

Respiratory tract infections are the main problem. ICUs predominance of Gram Negative Bacilli in these infections may suggest the importance of exogenous sources. VAP and CAUTI rates are also high, diminish its incidence is priority for the national program.

## Disclosure of interest

None declared.

